# Electrically Conductive TPU Nanofibrous Composite with High Stretchability for Flexible Strain Sensor

**DOI:** 10.1186/s11671-018-2499-0

**Published:** 2018-03-27

**Authors:** Lu Tong, Xiao-Xiong Wang, Xiao-Xiao He, Guang-Di Nie, Jun Zhang, Bin Zhang, Wen-Zhe Guo, Yun-Ze Long

**Affiliations:** Collaborative Innovation Center for Nanomaterials and Devices, College of Physics, Qingdao University, Qingdao, 266071 China

**Keywords:** Electrospinning, Conductive nanofibrous composite, Strain sensor

## Abstract

Highly stretchable and electrically conductive thermoplastic polyurethane (TPU) nanofibrous composite based on electrospinning for flexible strain sensor and stretchable conductor has been fabricated via in situ polymerization of polyaniline (PANI) on TPU nanofibrous membrane. The PANI/TPU membrane-based sensor could detect a strain from 0 to 160% with fast response and excellent stability. Meanwhile, the TPU composite has good stability and durability. Besides, the composite could be adapted to various non-flat working environments and could maintain opportune conductivity at different operating temperatures. This work provides an easy operating and low-cost method to fabricate highly stretchable and electrically conductive nanofibrous membrane, which could be applied to detect quick and tiny human actions.

## Background

Nanofibrous membranes have stimulated enormous attention for their outstanding chemical and physical performance, such as high specific surface area, high porosity, elasticity in surface functions, and outstanding mechanical performance. These excellent properties make the polymer nanofibrous membrane a potential material in many fields such as tissue template [1–4], protective clothing application [5], drug deliver [6–8], and electronic devices [9, 10]. And these applications usually require highly stretchable devices which could be applied to irregularly shaped objects. There are a lot of approaches to get a nanofibrous membrane, such as template synthesis [11, 12], ultrasonic irradiation synthesis [13], nanoprinting [14], and electrospinning [15]. Among these methods, electrospinning is a simple, low-cost, and convenient method to fabricate nonwoven membranes, and it is portable to generate nanofibrous membrane in lab. The electrospun micro/nanofibers display a variety of outstanding properties, such as large surface area, high length/diameter ratio, flexible surface functionality, and superior mechanical performance.

To acquire electrical conductivity, conducting polymers and carbon series semiconductor materials are often used as functional elements in the fabrication of membrane. Polyaniline (PANI) is a kind of conductive polymer with high conductivity and is easily to be polymerized. However, the strong polarity, which induces high conductivity, leads to poor elasticity of PANI [16]. Thermoplastic polyurethane (TPU), as one of the high-elasticity materials, is characterized with high elasticity, low-temperature flexibility, and abrasion resistance [17]. The combination of TPU and PANI can make up the disadvantage of PANI, and the strong polarity of PANI makes efforts to combination. Besides, the TPU membrane obtained by electrospinning is of high elasticity, high stretchability, low cost, and light weight. In situ polymerization exhibits a good way to combine TPU membrane and PANI together. As for flexible strain sensor and stretchable conductor, which could be applied in wearable electronic devices, elasticity and conductivity are essential, so we choose TPU and PANI as raw materials to fabricate nanofibrous composites. In this paper, highly stretchable and electrically conductive TPU nanofibrous membrane based on electrospinning for flexible strain sensor and stretchable conductor has been fabricated via post-processing strategies. The PANI/TPU composite sensor could sustain a maximum tension of 165%, and the conductivity of our strain sensor can be calculated to be about 7.5 × 10^−3^ S cm^−1^. Meanwhile, the composite displays good stability and durability. Moreover, the composite could be applied to various non-flat working environments and could maintain almost good conductivity at different operating temperatures. This work provides a facile operating and low-cost method to fabricate highly stretchable and electrically conductive nanofibrous membranes, which have potential applications in flexible strain sensors and stretchable conductors for wearable devices.

## Experimental

### Preparation of PANI/TPU Nanofibrous Membrane

There were three steps to prepare PANI/TPU membrane. The first step was to obtain TPU nanofibrous membrane via electrospinning. 2.4 g TPU was dissolved in 8.8 g *N*,*N*-dimethylformaminde (DMF) and 8.8 g tetrahydrofuran (THF) to prepare a precursor solution and then stirring the mixture thoroughly for 5 h until it turned into a homogeneous solution. The electrospinning process was carried out with a spinning distance (between the needle and collector) about 10~12 cm, a high voltage (supplied by a high-voltage DC power, DW-P303-1ACFO, Tianjin Dongwen) about 12 kV, and a feeding rate of the solution (maintained by a syringe pump, LSP01-1A, Baoding Longer Precision Pump Co., China) about 15 μl min^−1^. Moreover, to get a uniform thickness nanofibrous membrane, a roller was used as a collector. Compared with the traditional collector like aluminum foil, the thickness of the membrane was more uniform from the edge to the middle. After getting the TPU membrane, the next step was the polymerization of PANI. Firstly, 4.6 g ammonium persulfate (APS, *M*_w_ = 228.20) was added into 50 ml deionized (DI) water to make up the solution A and 1.875 g aniline (*M*_w_ = 93.13) and 2.54 g sulfosalicylic acid (SSA, *M*_w_ = 254.22) were dissolved into 50 ml DI water to obtain solution B. After stirring for 30 min at room temperature, the TPU membrane(10 cm × 10 cm) was submerged into solution B, and then, solution A was slowly added to B to ensure the intensive mixing. After standing in refrigerator for 12 h at 275 K, the membrane was taken out from the final solution and washed with DI water. With the polymerization reaction of aniline, the color of the mixture changed from canary yellow to deep green and the membrane changed from white to deep green. Finally, the PANI/TPU nanofibrous membrane was obtained after drying for 48 h at room temperature.

### Sensor Assembly

**Fig. 1:**
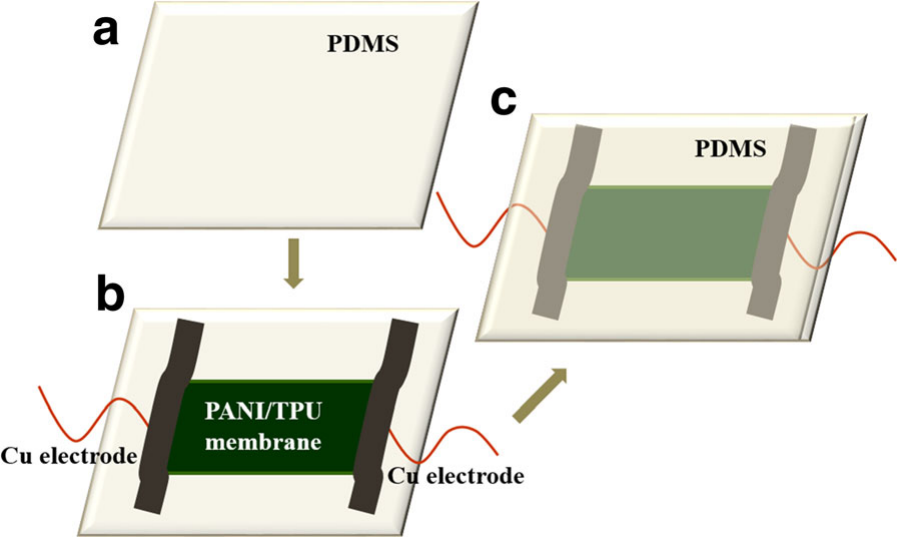
Schematic illustration of the sensor assembly process

As shown in Fig. 1, the highly stretchable and conductive nanofibrous TPU composite-based strain sensors were assembled by sandwiching a piece of composite membrane (1 cm × 2 cm × 0.05 cm) with two PDMS films (which were used to prevent the nanofibrous membrane from being destroyed, 1.5 cm × 3 cm × 0.05 cm), and two copper wires were fixed by silver pastes as electrodes. The width of the membrane was 15 mm, and the distance between the two copper wires was 1.5 mm.

The final nanofibrous membrane was characterized by an optical microscope (Olympus BX51), a scanning electron microscope (SEM, DB235 FEI), and a Fourier transform infrared spectroscope (FTIR, Thermo Scientific Nicolet iN10). The strain-stress curves of the twisted fibers were obtained by a dynamical mechanical analyzer (Q-800, TA Scientific). Electrical properties were tested by a Keithley 6485 high-resistance meter system at room temperature and a physical property measurement system (PPMS, Quantum Design).

## Results and Discussion

### Characterizations of Nanofibrous Membrane

**Fig. 2:**
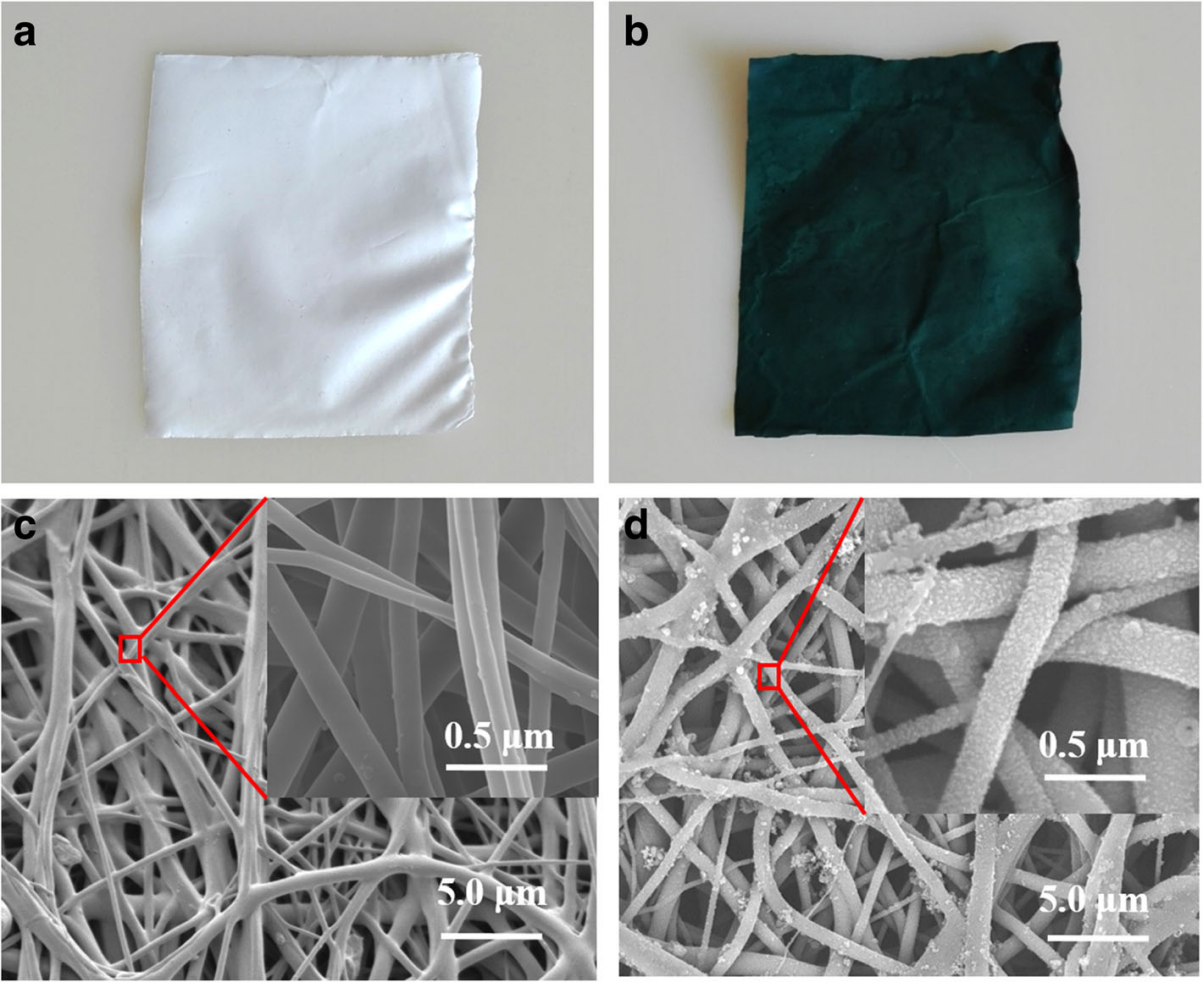
Morphology and structure of TPU and PANI/TPU membrane. **a**, **b** Optical images of pure TPU nanofibrous membrane and PANI/TPU nanofibrous membrane. **c**, **d** SEM images of pure TPU nanofibrous membrane and PANI/TPU nanofibrous membrane

Pure nonwoven TPU mat has high elasticity. After in situ polymerization of PANI, the composite is of good conductivity, good stretchability, and high elasticity. These properties meet the requirements of stretchable devices, such as wearable devices [9, 10], skin-like sensor [9], and microfluidic device [18]. After the polymerization, the nanofiber membrane changes from white to deep green (Fig. 2a, b). From the SEM images of the membranes, we can see that the surface of PANI/TPU fibers (Fig. 2d) is covered with PANI particles (Fig. 2d).

**Fig. 3:**
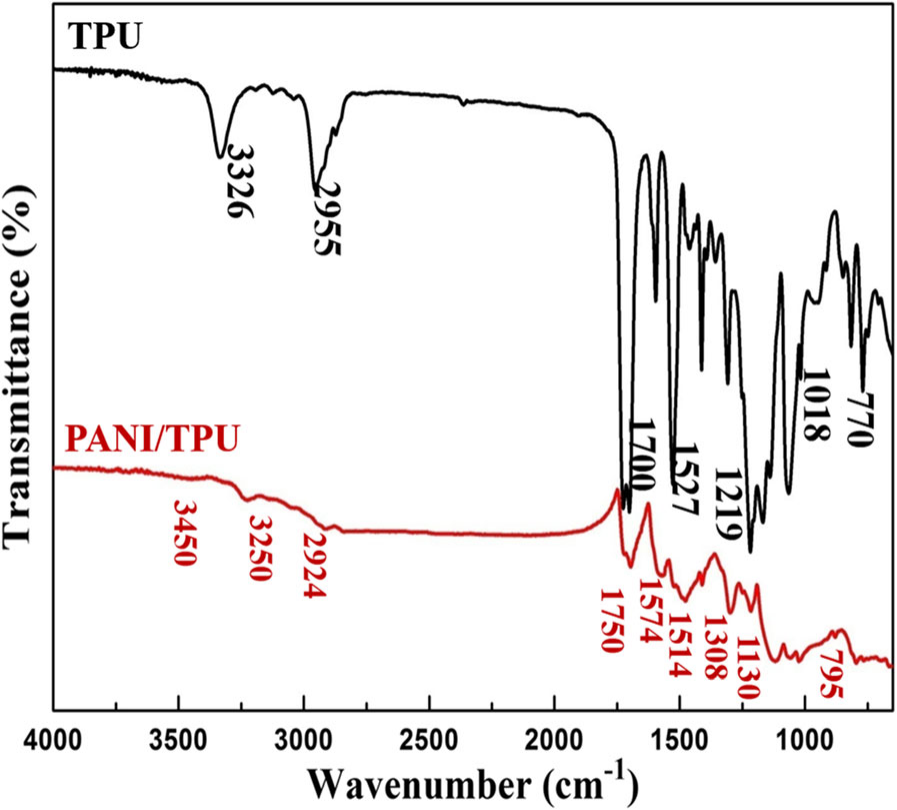
FTIR spectra of TPU and PANI/TPU nanofibrous membranes

Figure 3 shows the FTIR spectra of pure TPU and PANI/TPU nanofibrous membrane. The FTIR spectra of TPU indicates the N–H absorption of carbamic acid ester at 3326 and 2955 cm^−1^. The bands at 1700 and 1527 cm^−1^ are consistent with dissociative C=O of amino of carbamic acid. In the spectra of the PANI/TPU, the new 3250 cm^−1^ absorption band is assigned to N–H stretching vibration of –C_6_H_4_NHC_6_H_4_– of PANI, and the C=C vibration of aromatic appears at 1514 cm^−1^ [19, 20]. These bands indicate the existence of PANI.

### Stretchability and Sensitivity Test

**Fig. 4:**
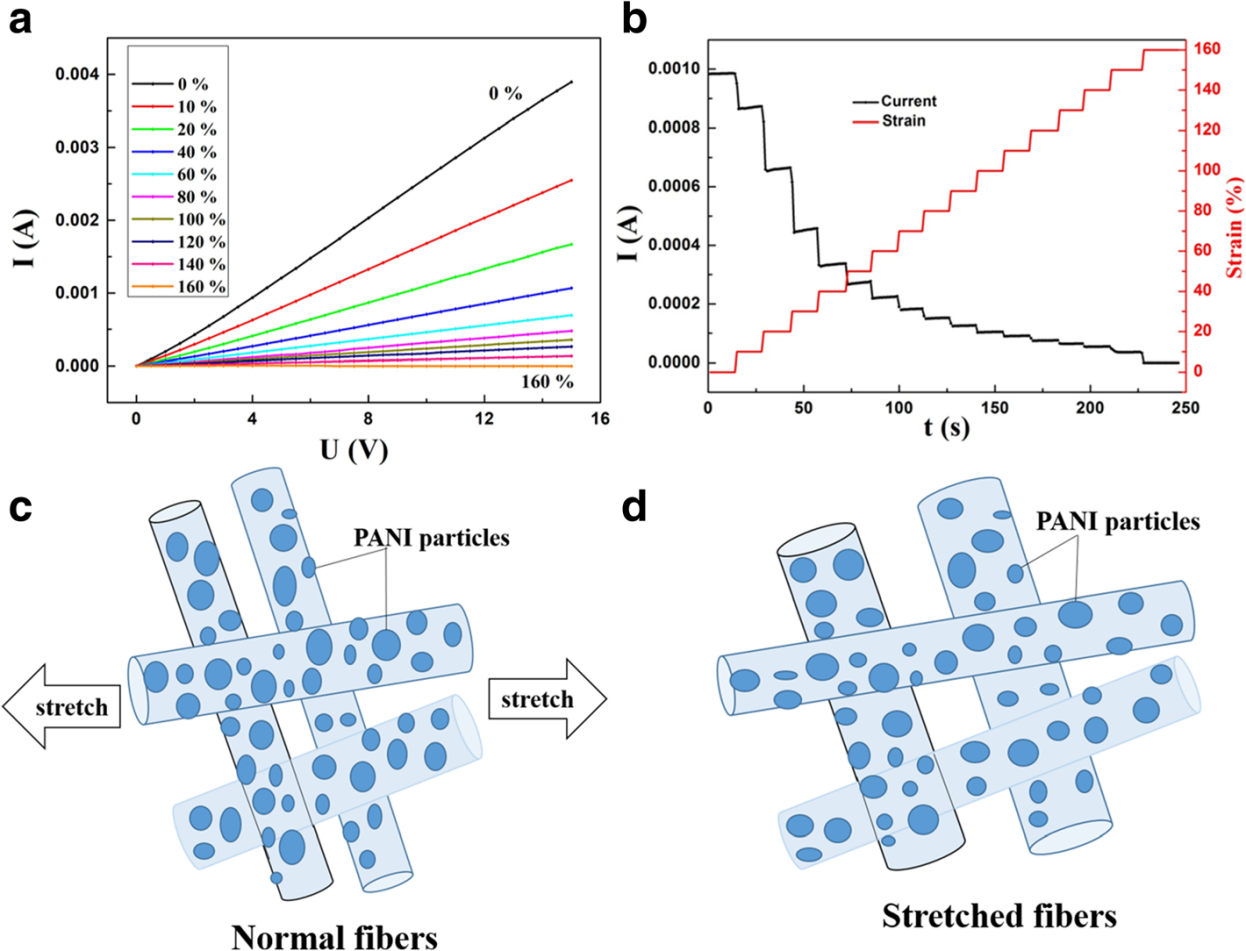
Stretchability test and schematic diagram of the PANI/TPU membrane sensor. **a** *I*-*V* curves of PANI/TPU membrane under different strains. **b** Current responses of PANI/TPU membrane to different strains under a fixed bias of 5 V. **c** Fibers under no strain. **d** Fibers under strain

The composite nanofibrous membrane is characterized with good elasticity and high stretchability, and its conductivity changes with stretching, namely, the PANI/TPU nanofibrous membrane could be used in strain sensors. Figure 4a shows the *I*-*V* characteristics of the PANI/TPU sensor with different tensions. The *I*-*V* curves of PANI/TPU sensor have good linear relation. From the *I*-*V* characteristics of the sensor, it can be seen that the PANI/TPU sensor can tolerate a strain up to 165%. Notably, the current decreases gradually with increasing the strain of the sensors. Figure 4b shows the current response of a continuous strain ranging from 0 to 160% of the PANI/TPU sensor. From the current response to the continuous strain, we can see that the sensor has good stability. The PANI/TPU nanofibrous membrane possesses better mechanical character than a reported patterned PVDF nanofibrous membrane [21]. The working principle of the fabricated Ag/alginate nanofibers for pressure sensor is schematically illustrated in Fig. 4c, d.

**Fig. 5:**
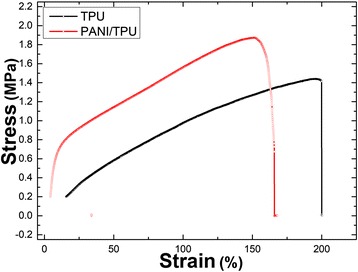
Strain-stress curves of TPU and PANI/TPU nanofibrous membranes

In addition to these electrical properties, the mechanical properties of pure TPU and PANI/TPU nanofibrous membranes have also been studied, as the stress-strain responses displayed in Fig. 5. From the stress-strain curves, we know that pure TPU membrane can be stretched up to about 200% and the PANI/TPU membrane is about 165%. The full stress-strain curve of PANI/TPU nanofibrous membrane can be ranged into three regions: (1) 0–19% is the elastic region, where the deformation is recoverable; (2) 19–140% is the plastic region, in which the deformation would never be recovered; and (3) the third region is the elongation at break which is about 165%. From Fig. 5, we can see that the tensile strength of PANI/TPU membrane increased to 1.93 MPa, due to the presence of PANI which is brittle in nature, but a decrease in strain at a break of 165% compared with that of TPU nanofibrous membrane [22].

**Fig. 6:**
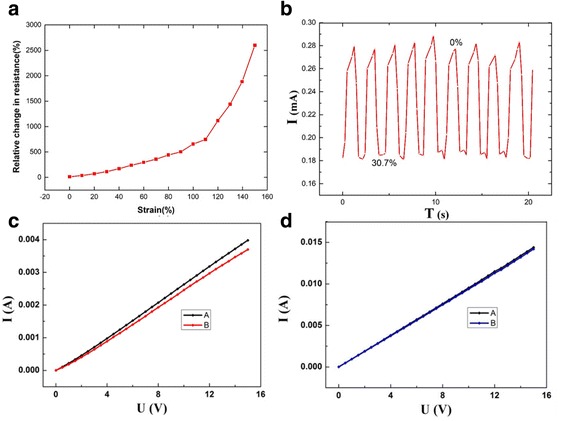
Stability and durability test of the PANI/TPU membrane sensor. **a** Relative changes in resistance of the PANI/TPU membrane sensor under different strains. **b** Stability test under a fixed strain of 30.7%. **c** A is the *I*-*V* curve at the initial stage, and B is the *I*-*V* curve after 100 times stretching to 30.7% and placed for 24 h. **d** A is the *I*-*V* curve at the initial stage, and B is the *I*-*V* curve after 1000 times bending and placed for 24 h

As is well known, the gauge factor (GF) is a typical performance index of a strain sensor, and it is defined as (d*R*/*R*_off_)/*ɛ* which means the ratio of relative change in electrical resistance (d*R*/*R*_off_) to the mechanical strain *ɛ*. It exhibits the sensitivity change of the sensor to tension. *R*_off_ is the resistance of the sensor in the formula, and d*R* is the change of the resistance of the sensor [18, 21]. Figure 6a shows the relative change of resistance of the sensors. When the sensor was stretched to 120%, fibers began to break. The breakages result in the largely increased distance between conductive particles, and thus, the resistance has a great change from 120 to 150%. Figure 6a indicateds that the strain rate of PANI/TPU membrane varies from 0% to 150%. The GF is about 6.7252 from 0 to 120% and about 49.5060 from 120 to 150%. The data obtained from the experiments show that the PANI/TPU sensor has good sensitivity. While compared with other reports, the GF is lower than some advanced ultrathin silicon-based strain sensors (GF is about 200), PEDOT:PSS/PVA films [23], and those strain sensors which are fabricated by single inorganic nanotube and nanowire [24–26]. However, the sensitivity is better than PANI/PVDF sensors (GF is about 1) [21].

Only these properties are not enough. A good strain sensor should be equipped with good stability and durability which implies the sensor can work for a long time without any significant regression after different elastic deformations. To measure the stability, we investigated the response-recovery curve under a fixed strain of 30.7%, and the result is shown in Fig. 6b. Here, the current decreases with tensile strain and the current almost recovered to the initial value. And then, the curve could repeat the same circle under 30.7% mechanical pressure which suggests that our sensor had a good repeatability. In practical applications, durability is an important parameter [18]. To access the endurance of the sensor, we investigated the output signals under 100 times cycling stretching and placed it for 24 h at room temperature. The results are shown in Fig. 6c. Curve A represents the original *I*-*V* characteristic of the sensor without any stretch, and curve B is the *I*-*V* characteristic of the sensor which was stretched for 100 times and put for 24 h. The function mechanism of the conductivity response may be due to the rupture and falling off of the PANI cluster or separation of PANI particles which makes the conductivity decrease. Figure 6d shows that the *I*-*V* characteristic after 1000 times of bending almost has no change compared with the initial value. The results indicate that the sensor is characterized with good durability.

**Fig. 7:**
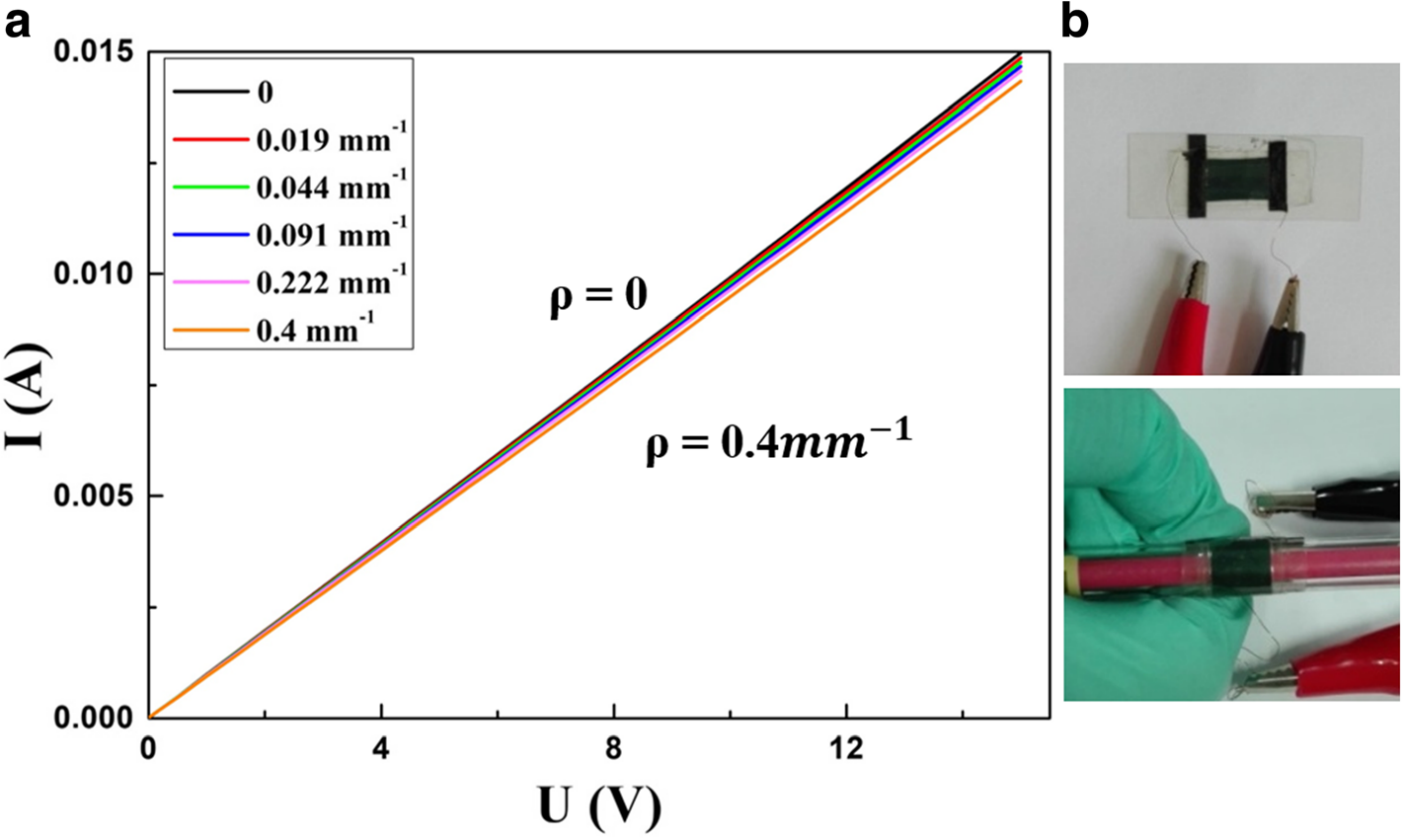
**a** *I*-*V* curves of the PANI/TPU membrane sensor under different curvatures. **b** Optical images during the test of *I*-*V* characteristics under different curvatures

**Fig. 8:**
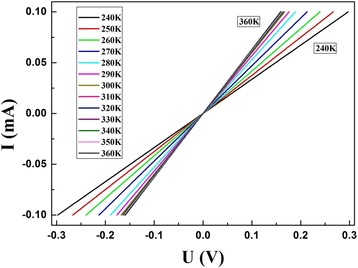
*I*-*V* curves of the PANI/TPU membrane sensor under different temperatures

A good sensor should have little response to the change of environment. In addition to tensile force, as wearable device, it should also be freely bent. Herein, to demonstrate the bendable characteristic, we detect its output signals under different curvatures. To test the bendability of the sensor, the *I*-*V* characteristics are estimated when it is fixed on items with different curvatures. As depicted in Fig. 7a, just small changes appear when the curvature alters from 0 to 0.4 mm^−1^, which suggests that the sensor could be adapted to various non-planar working environments. Besides, to determine temperature drift, we tested the *I*-*V* characteristics of the sensor under different temperatures. Figure 8 displays the *I*-*V* curves under different temperatures. When the temperature changes from 240 to 300 K, the resistance has a modest and regular decrease from 2.9697 to 1.6025 kΩ, and notably, there only exists a tiny disturbance (0.0556 kΩ) when temperature changes from 300 to 360 K. The sensor could maintain good conductivity. The result indicates that although the electric conductivity changes slightly, the sensor could keep good conductivity under different temperatures. The results confirmed the sensor could work normally under different ambient temperatures. Figure 7b shows the device for measuring the currents under different curvatures of the sensor.

### Application in Finger Bending-Release Detection

**Fig. 9:**
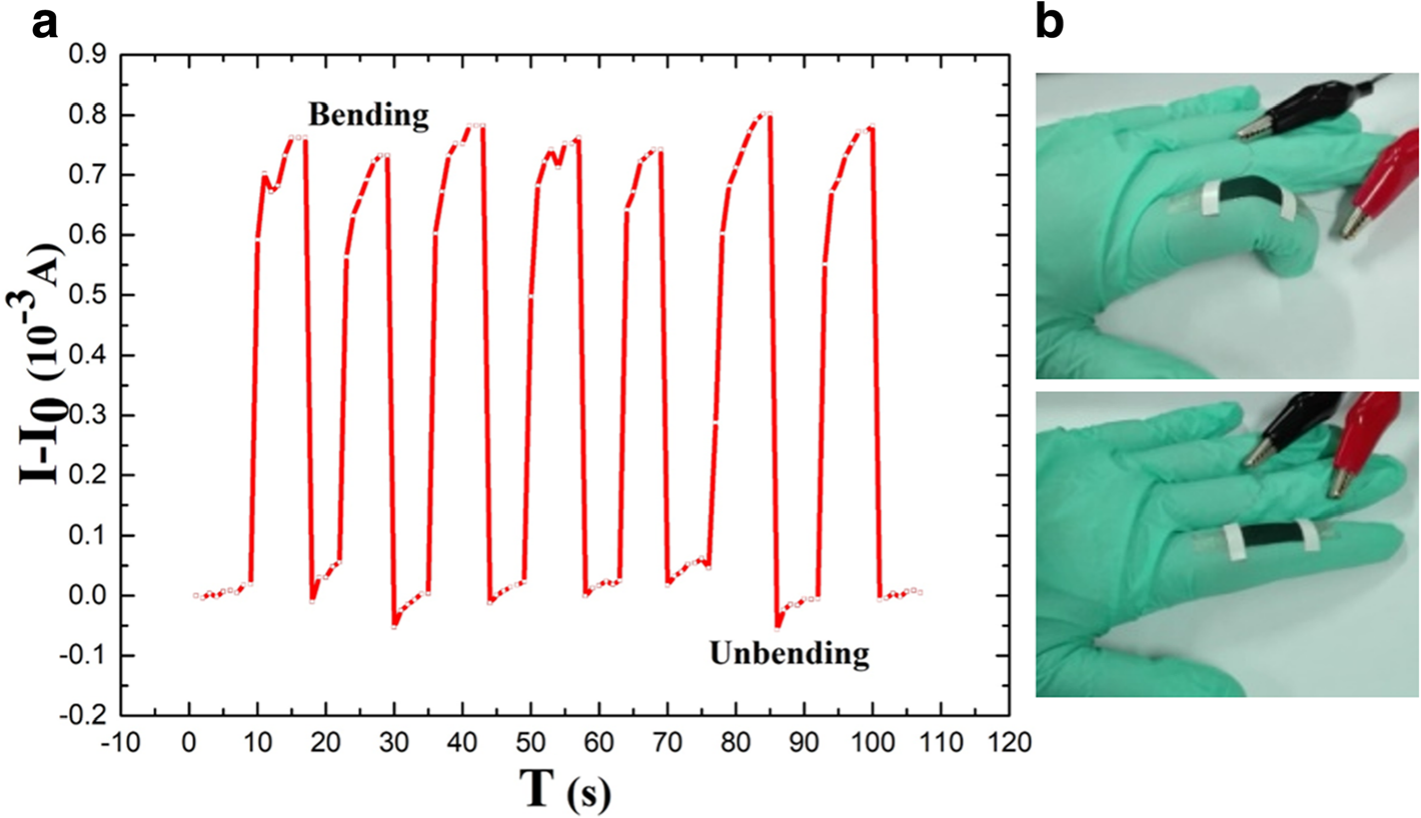
**a** Current responses of the finger motion and photographs of the wearable PANI/TPU membrane sensor. **b** Optical images of the finger motion test

We utilized finger motion to simulate the movement of human. Figure 9a reveals the typical response curve of the sensor. We tested for nearly 2000 times of finger bending and only seven cycles are shown, and Fig. 9b is the photograph of sensor for detection of finger movements (with a strain of 1%). The electric transport of the sensor was affected by the external force. When finger-bended, the currents jumped to maximum, the maximum remained while finger kept bending, and then back to its original value after unbending. From the time-resolved current response, it can be seen that the sensor has a good response and recoverability to external force. Nowadays, an increasing interest surrounds wearable biosensors [27], which can be used to detect a range of bio-signals such as blood pressure [28] and wrist pulses [29] and can be used to monitor joint and muscle motion [30]. There are many reports about this kind of sensors which put them into smart clothes or attach it to the skin directly to detect human motion [9, 30–32], because of its low cost, light weight, and good sensitivity [29]. Herein, based on the above-mentioned test results, our strain sensors exhibit potential applications in wearable devices. The good sensitivity, light-weight, and low-cost properties of the sensor demonstrate there are many potential applications, such as in health care and multi-functional intelligent room [9, 10, 32].

**Fig. 10:**
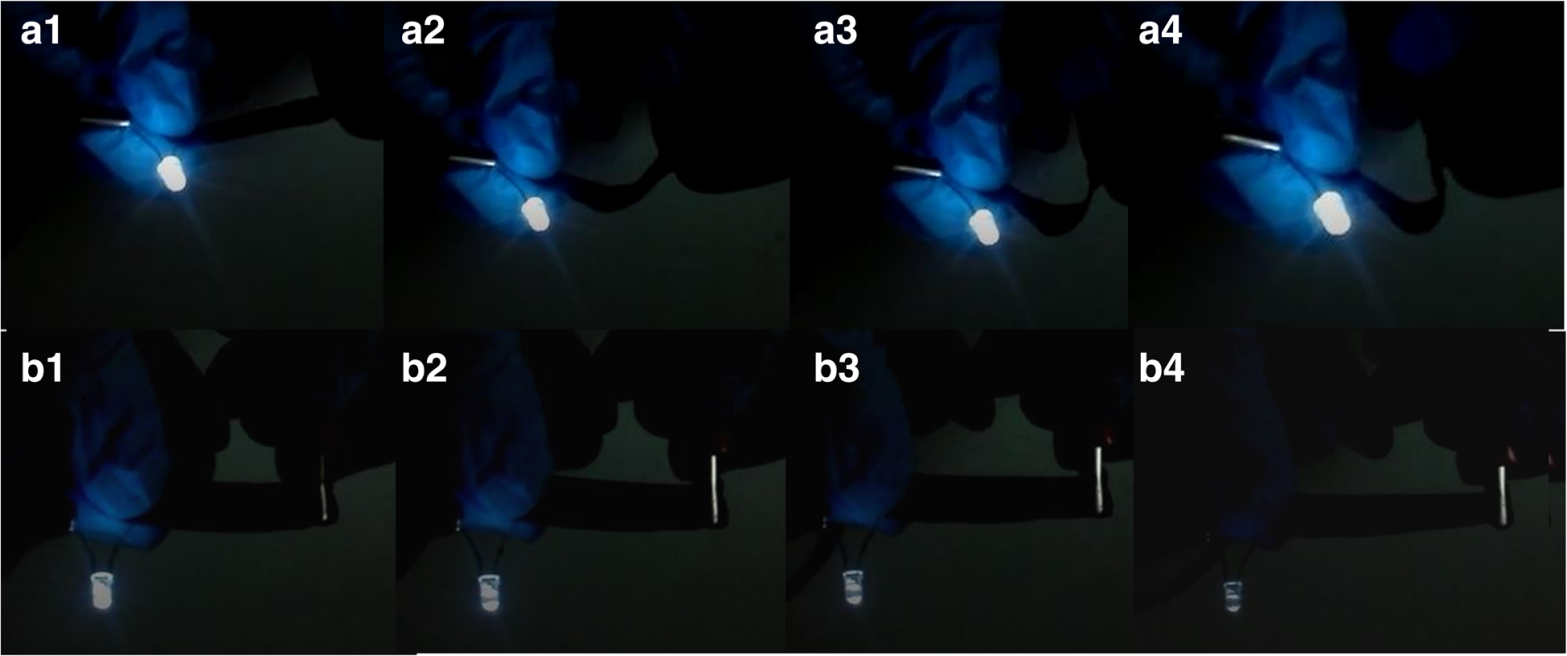
Flexible conductor of the PANI/TPU membrane in closed circuit. **a** Self-flashing LED could give out light normally when the flexible conductor of PANI/TPU membrane was under different curvatures. **b** Self-flashing LED dimming with tensile strain of PANI/TPU membrane

The sensor does not have to rely on complicated electrical property measure system, a simple self-flashing LED was used to fulfill the character task. Figure 10a_1_–a_4_ shows that the LED could give out light normally when the flexible conductor of PANI/TPU membrane is under different curvatures (0, 0.1, 0.05, and 0.033 mm^−1^, respectively). Figure 10b_1_–b_4_ exhibits a more significant light change with stretching (0, 20, 40, and 60%, respectively). The brightness of the LED dims with the increasing strain of PANI/TPU membrane. Through brightness variations of the LED light, we can know the status of the sensor, which is applicable in situations where space limitation exists.

The sensor has sensitivity and good stretchability, and Fig. 10 indicates the PANI/TPU nanofibrous membrane could be used as a flexible conductor which has the potential to be applied to flexible screen and may be attached to clothing to detect human health [33].

## Conclusions

In summary, we fabricate highly stretchable nanofibrous PANI/TPU strain sensor via electrospinning. The sensor based on PANI/TPU nanofibrous membrane can detect and withstand a strain from 0 to 165% with fast response and excellent stability. In addition to high stretchability, it shows good qualities in the durability and stability under different ambient environments. Moreover, because of the fast and repeatable response to tensile force and finger motions, the simple device could be applied to detect quick and tiny human actions. Meanwhile, thanks to the high conductivity, it could be used as flexible conductors for electronic components. This work provides a facile method to fabricate highly stretchable and conductive nanofibrous membrane with characteristics of fast dynamic motion-sensing abilities, high stability, and cheap fabrication.

## Abbreviations

DI water: Deionized water
PANI: Polyaniline
TPU: Thermoplastic polyurethane
